# Maternal exacerbating and protective factors that shape the prevalence and severity of child attention-deficit hyperactivity disorder: a narrative review

**DOI:** 10.3389/fpsyt.2025.1577707

**Published:** 2025-05-29

**Authors:** Corinne Thorsheim, Sana Khan, Ye Lu, Robert P. Kauffman

**Affiliations:** ^1^ Rocky Vista University College of Osteopathic Medicine, Parker, CO, United States; ^2^ Department of Obstetrics and Gynecology, Texas Tech University Health Sciences Center School of Medicine, Amarillo, TX, United States

**Keywords:** attention-deficit hyperactivity disorder (ADHD), maternal genetic factors, maternal environmental factors, maternal exacerbating factors, maternal protective factors, maternal psychopathologies, developmental outcomes

## Abstract

Attention deficit hyperactivity disorder (ADHD) is a widely prevalent neurodevelopmental disorder that affects millions of children and adolescents in the U.S. Despite the growing number of diagnoses, the maternal exacerbating and protective factors influencing ADHD symptom severity in offspring remain largely understudied. This narrative review examines the interplay between genetic and epigenetic factors, focusing on specific single nucleotide polymorphisms, polygenic risk scores for ADHD and comorbidities, mitochondrial DNA haplotypes, and X-linked inheritance. Key epigenetic influences include maternal gestational weight gain, young parental age, parental gene–psychopathology interactions, shared genetic loci between maternal educational attainment and ADHD, maternal prenatal stress, maternal hostility and maltreatment, postnatal household chaos, and parenting styles, all which shape ADHD symptom severity in the context of genetic predispositions. Importantly, the positive effects of elevated socioeconomic status and positive parenting on symptom severity may also be influenced by maternal genetic factors, representing an avenue for further research. The maternal environmental factors associated with ADHD in offspring, such as *in utero* acetaminophen exposure, maternal diet, vitamin D deficiency, and exposure to toxins, particularly from maternal smoking, are highlighted. Ultimately, this review seeks to uncover the combined impact of maternal environmental and existing factors on underlying parental genetics—a critical aspect often overlooked in existing studies. Clinical implications are also addressed, particularly concerning differences in beta and theta wave activity and variations in cerebral blood flow in the dorsolateral prefrontal cortex between children with ADHD and those with comorbid autism spectrum disorder (ASD). By understanding these multifaceted factors, especially maternal contributions, alongside emerging clinical diagnostic strategies, better targeted interventions can be aimed at enhancing treatment efficacy and improving long-term outcomes for children with ADHD.

## 1 Introduction

Attention deficit hyperactivity disorder (ADHD) is one of the most prevalent neurodevelopmental disorders in children, and its incidence has increased significantly in recent years, underscoring the urgency of studying this condition. Previous research from 2020 estimated that the incidence of ADHD was 5% among children and 2.5% among adults (1). However, the most recent CDC data from March 20, 2024, revealed a marked increase, with 11.3% of U.S. children aged 5–11 years being diagnosed with ADHD between 2020 and 2022 (2). This condition manifests most prominently in childhood and adolescence, with notably higher diagnosis rates in boys (14.5%) than in girls (8%), indicating a substantial gender disparity (2).

The implications of ADHD are profound, as approximately 60% of affected individuals continue to experience symptoms into adulthood (3, 4). Diagnosis requires clear evidence of substantial interference with developmental functioning, as outlined in various clinical guidelines (4, 5). ADHD is further categorized into three primary subtypes: predominantly inattentive, predominantly hyperactive/impulsive, and combined presentation, each defined by a specific symptom profile essential for accurate identification (see **Table 1**).

**Table 1:** Diagnosis and symptoms of ADHD.

Subtype	Inattentive	Hyperactive/Impulsive	Combined
**List of Symptoms** ^a^	• Careless mistakes • Difficulty sustaining attention • Not listening when spoken to • Not follow–through on instructions and fails to finish tasks • Difficulty organizing tasks • Avoiding tasks requiring mental effort • Losing things necessary for tasks • Easily distracted • Forgetful in daily activities	• Fidgeting or tapping hands • Difficulty remaining seated • Running or climbing excessively in inappropriate situations • Difficulty playing quietly • Often “on the go” or acts “driven by a motor” • Talking excessively • Blurting out answers • Difficulty waiting turn • Interrupting others	The criterion for both the inattentive and hyperactive/impulsive subtypes were met for greater than 6 months.
**Diagnostic criteria** ^b^	• Symptoms present for at least 6 months before 12 years old.^c^ • ≥ 6 symptoms in ≥ 2 settings. • These symptoms are not solely a manifestation of oppositional defiant, hostility, defiance, or lack of understanding of tasks or instructions. • Significant social, academic or occupational impairment present inconsistent with developmental level. • Levels: minimal, intermediate, severe impairment.^d^ • Specify if in partial remission, full criterion was previously met, but now fewer than the full criteria have been met for greater than 6 months with continued impairment.^e^ • The symptoms do not occur exclusively during schizophrenic or other psychotic disorder episodes and are not better explained by another mental disorder.		

a. These diagnostic symptoms have been simplified for clarity.

b. Diagnostic criteria adapted from the DSM–5–TR (Diagnostic and Statistical Manual of Mental Disorders, Fifth Edition Text Revision).

c. At least five symptoms are required for older adolescents and adults (aged 17 years and older).

d. The severity level is based on the number of symptoms present in excess of the minimum required to make the diagnosis and is ranked on the basis of the impact on social and/or occupational functioning.

e. Sixty percent of ADHD patients diagnosed in childhood experience persistent impairment into adulthood (3).

*For a complete description of symptoms and additional diagnostic criteria, please refer to the DSM–5*–TR* (5) *and Kaplan & Sadock's Concise Textbook of Clinical Psychiatry, 5e* (3).

Since the publication of the most recent DSM-5-TR definition in 2022 (5), ADHD diagnoses have continued to rise, largely owing to a broader understanding of its heterogeneous symptomology across age groups, genders, and socioeconomic contexts (6). Recent research has increasingly examined the often-overlooked nuances of this disorder, revealing gender-based differences. Studies indicate that females typically experience greater intellectual impairment (7) and internalizing behaviors, which encompass distress and negative emotions directed inward, such as anxiety, disorganization, distraction, and forgetfulness (8, 9). In contrast, males are more likely to exhibit externalizing behaviors, which are directed outward and include hyperactivity, impulsivity, and disorderly conduct (10). In addition to these correlated sex-based comorbidities, which can be broken down by subtype, there are also developmental outcomes and comorbidities associated with the different subtypes of this disorder (see **Table 2**). Furthermore, the current male-to-female diagnosis ratio of this disorder is approximately 3:1 in childhood, with a notable increase to a ratio of 1:1 in adulthood (16). This variability highlights the need to examine how social, environmental, and genetic factors interact collectively to shape the development and severity of ADHD, which can have life-long impacts.

**Table 2:** Comorbidities, developmental outcomes, and behavioral patterns in ADHD patients.

Subtypes	Inattentive ^b^	Hyperactive/Impulsive ^c^	Combined
**Main comorbidities** ^a^	• Anxiety Disorders • Major depressive disorder • Learning Disabilities • Mood Disorders • More prevalent in females	• Oppositional Defiant Disorder • Conduct Disorder • Intermittent explosive Disorder • More prevalent in males	Common comorbidities from both categories, notably, autism spectrum disorder
**Developmental Outcomes** ^d^	• Higher risk of academic underachievement • Social isolation reported, especially in females • Self–esteem challenges in adulthood	• Behavioral issues in school settings • Higher likelihood of legal issues in adolescence	• Superior behavior, moods, social skills and academic performance outcomes most notable in this subtype for combination pharmacotherapy + psychosocial therapy ^e^ • Individuals with lower socioeconomic status shown to exhibit significantly worse outcomes and lost to follow–up compared to other subtypes ^f^
**Externalizing vs. Internalizing Behaviors** ^g^	• Emotional distress • Frustration • Anxiety • Sadness • Somatization • More prevalent in females	• Peer victimization (aggression toward others) • Victimless forms (such as substance abuse) • More prevalent in males	Combination of both internalizing and externalizing behaviors in females and males, respectively.

a. Comorbidities are generalized and may not apply to every individual.

b. Data were adapted from *the Impact of Internalizing Symptoms on Impairment for Children with ADHD: A Strength–Based Perspective* (11).

c. Data were adapted from the *Encyclopedia of Personality and Individual Differences: Externalizing Behavior* (12).

d. Children with severe ADHD symptoms are at risk of experiencing substance abuse, interpersonal difficulties, conduct problems, and delinquency as well as alcohol, nicotine, marijuana, cocaine and other unspecified drug–use disorders regardless of the specific subtype (13).

e. The data were adapted from the landmark study: *the MTA Cooperative Group. The Multimodal Treatment Study of Children with ADHD* (14), which involved over 500 children diagnosed with DSM–IV ADHD, established the benefit of combining medication with psychosocial interventions (15).

f. Data were adapted from the follow–up study after the original 1999 MTA study. *MTA at 8 years: Prospective Follow–Up of Children Treated for the Combined Type of ADHD in a Multisite Study* (13).

g. Data were adapted from *Gender differences in ADHD: a meta–analysis and critical review* (6).

Existing research and reviews have focused predominantly on genetics, environmental factors, psychopathology, and protective influences in isolation. Few studies have taken a holistic approach to integrate these elements, and many fail to adequately explore how maternal environmental exposures interact with genetic expression. This review not only advances the latest genetic literature on ADHD but also integrates new insights into maternal-specific mitochondrial DNA inheritance, X-linked expression, and maternal gene-psychopathology interactions, all while acknowledging the limited but credible paternal-specific findings associated with this disorder. Furthermore, while a significant body of previous studies has highlighted *in utero* influences, this review broadens its scope to encompass exposures throughout all pre-pregnancy, prenatal, perinatal, and postnatal periods.

This narrative review aims to provide a comprehensive synthesis of the intricate ways in which maternal-specific influences affect ADHD. It encompasses a variety of elements, including genetics, epigenetics, environmental factors, and clinical manifestations. Maternal environmental influences are examined across all relevant periods, highlighting their potential to modulate ADHD severity in genetically predisposed children and addressing a critical gap in the literature. By evaluating these factors collectively, we recognize that each may contribute to the risk and severity of ADHD while also suggesting the potential for protective effects through appropriate intervention. The objective is to offer a holistic understanding of how these interconnected elements shape the onset and severity of ADHD in offspring.

## 2 Review strategy

### 2.1 Inclusion/exclusion criteria

A narrative review format was chosen because of the diverse range of correlations and contributing maternal mechanisms associated with ADHD development in offspring. Given the heterogeneity of study designs and methodologies in the literature, a qualitative synthesis was deemed most appropriate. Literature searches were primarily conducted via PubMed, Scopus, Google Scholar, and Elsevier, with sources collected in December 2024. Only English-language publications examining parental (primarily maternal) influences on ADHD in offspring were included. The search terms are listed in **Table 3**.

**Table 3:** Search terms.

ADHD contributing maternal factors ADHD AND genetic predisposition ADHD AND environmental influence ADHD AND maternal genetic predisposition ADHD AND maternal protective factors maternal psychopathologies AND offspring ADHD genetics AND nurture AND ADHD maternal genetics AND nurture impacting offspring ADHD maternal psychopathologies AND environmental influence impacting the behaviors and severity of offspring ADHD the relationship between maternal genetics AND nurture shaping offspring ADHD maternal and child ADHD AND household chaos AND systematic review maternal resilience AND decreased offspring ADHD AND systematic review maternal resilience AND maternal ADHD AND offspring ADHD AND/OR meta–analysis, systematic review, umbrella review child ADHD evocative effect AND maternal hostility AND symptom severity AND systematic review maternal age AND offspring ADHD AND systematic review AND/OR meta–analysis AND/OR umbrella review maternal resilience AND/OR risk avoidance AND child ADHD AND/OR meta–analysis mitochondrial DNA AND offspring ADHD AND/OR systematic review, meta–analysis or umbrella review maternal hostility AND offspring ADHD AND meta–analysis maternal heavy metal exposure AND offspring ADHD AND/OR systematic review maternal low vitamin D AND offspring ADHD AND/OR systematic review, meta–analysis, and umbrella review ADHD genes AND psychiatric disorders AND PRS AND systematic review genes AND ADHD AND psychiatric disorders AND PRS genes AND ADHD AND psychiatric disorders meta–analysis paternal influence AND offspring ADHD AND maternal influence birth–related hypoxia AND offspring ADHD systematic review OR meta–analysis OR umbrella review birth weight AND offspring ADHD systematic review low vitamin D AND offspring ADHD systematic review maternal SNPs AND offspring ADHD meta–analysis maternal prepregnancy obesity AND offspring ADHD AND systematic review preeclampsia AND offspring ADHD AND meta–analysis parental AND/OR paternal ADHD AND offspring ADHD paternal ADHD and child ADHD severity and prevalence paternal genetics AND offspring ADHD AND meta–analysis paternal AND epigenetic AND child ADHD AND meta–analysis paternal AND genetic AND child ADHD AND meta–analysis paternal depression AND child ADHD AND systematic, meta–analysis, and/or umbrella review father genes AND offspring ADHD prenatal intervention AND maternal factor AND offspring ADHD postnatal intervention AND maternal factor AND offspring ADHD interventions AND maternal factors AND child ADHD

A thorough screening and selection process was conducted to identify the most relevant, high-quality studies that examine maternal and paternal influences on the prevalence of ADHD in offspring, with an emphasis on maternal influence. This comprehensive review includes a range of research types—meta-analyses, observational studies, systematic reviews, and randomized clinical trials—with most studies published within the last 10 years, although a select number of high-quality, older studies were also included.

All observational studies included in this review were rated as either good or fair quality. When applicable, higher-quality meta-analyses, systematic reviews, clinical trials, or umbrella reviews were prioritized and used to support the observational studies utilized in our analysis. These observational studies were retained not only because they were bolstered by evidence from superior studies, but also due to their potential clinical relevance and their value in generating hypotheses for further exploration of this complex topic.

Last, the genetic subthemes of single nucleotide polymorphisms (SNPs), polygenic risk scores (PRS), and other genetic influences are grounded in more recent literature, with priority placed on studies published in 2023 and beyond, reflecting the growing importance of this emerging field.

## 3 Subsections

### 3.1 Genetics

#### 3.1.1 Single-nucleotide polymorphism-based heritability of ADHD

Recent research has shed light on the candidate single-nucleotide polymorphism (SNP) genes that correlate with the approximately 75% heritability of ADHD phenotypes (3). While large-scale twin studies have previously shown that siblings of children with ADHD (ages 5–17) are approximately nine times more likely to be diagnosed than control siblings are (17, 18), molecular genetic studies have since refined this understanding. These studies suggest that common genetic variants, particularly specific SNPs, contribute approximately 15–20% of the risk of developing ADHD (19). Genome-wide association studies (GWASs) have identified 27 significant loci potentially correlated with ADHD development, many of which are linked to genes involved in the dopaminergic neurotransmitter pathway, implicating 76 genes that are often upregulated during early brain development. Notable candidate genes include PTRF, FOXP1, FOXP2, MEF2C, SORCS3, and DUSP6 (20, 21). Mutations in these genes have been correlated with reduced cortical volumes and lower educational attainment (22), speech disorders, intellectual disabilities (23), and comorbidities with other psychiatric disorders (24, 25). Moreover, many of these mutations impact neurotransmitter homeostasis, particularly dopamine levels in the synaptic cleft (20). Given the biological relevance of dopamine in ADHD pathophysiology, numerous studies have explored these dopamine-related genetic pathways further, including DRD4, DRD5, DAT1,5-HTT/SLC6A4, HTR1B, NET1, SNAP-25, and BDNF (26, 27). While much attention has been given to dopaminergic dysfunction, recent imaging studies, including fMRI and SPECT, have also revealed altered activity in the prefrontal cortex and locus coeruleus (28), underscoring the importance of norepinephrine as a co-contributor to the impaired attention and impulse control common in this disorder (2).

#### 3.1.2 Genetic correlations and polygenic risk scores for child ADHD and comorbid phenotypes

Up to 70–80% of individuals with ADHD experience comorbid psychiatric disorders across their lifespan (29, 30). With advances in GWAS technology, researchers are increasingly able to identify shared high-risk genetic variants between ADHD and common comorbid psychiatric conditions, helping to illuminate which specific genes may contribute to both. For example, NRXN1 deletions have been linked to increased risk across ASD, ADHD, and schizophrenia, highlighting their pleiotropic effects on synaptic function and social-cognitive deficits (30). FOXP1 mutations, which disrupt the transcriptional regulation of neuronal pathways, are strongly associated with language impairment and intellectual disability, particularly in individuals with ASD (30). Similarly, ADGRL3 (formerly LPHN3) is implicated in ADHD through its influence on the dopaminergic signaling outlined previously, with downstream effects on impulsivity and attentional control. The growing body of evidence into these high-risk shared genetic loci offers promise in understanding the overlapping and divergent phenotypic traits across such disorders. Notable associations between ADHD and several lifetime psychiatric comorbidities include oppositional defiant disorder (ODD; ~45%), conduct disorder (CD; ~20%), personality disorders (up to ~60% in adults), major depressive disorder (MDD)/dysthymia (~40%), bipolar disorder (BD; ~15%), anxiety disorders (~35%), substance use disorders (SUD; ~25%), eating disorders (~10%), and autism spectrum disorders (29, 31).

Although these associations have been previously acknowledged broadly, advancements in genetic studies hold promise for clearly elucidating the maternal-specific genetic influences inherited by children with ADHD. Nonetheless, enhanced diagnostic precision has fostered a deeper understanding of which specific ADHD subtypes are most strongly correlated with common comorbidities. This progress also illuminates the shared long-term developmental outcomes associated with each specific subtype across genders (see **Table 3**).

Posttraumatic stress disorder (PTSD), substance use disorder (SUD), major depressive disorder (MDD) and autism spectrum disorder (ASD) have been shown to overlap strongly with ADHD via PRS and genetic correlation analyses (30). Cross-disorder analyses by the Psychiatric Genomics Consortium (PGC) built upon these earlier analyses as well as other GWAS findings by identifying shared risk loci across these disorders. An early analysis of five overlapping psychiatric conditions (ADHD, bipolar disorder, ASD, schizophrenia, and MDD) revealed four genome-wide significant loci near the genes ITIH3, AS3MT, CACNB2, and CACNA1C, with the first three showing relevance to ADHD (30, 32). A subsequent, larger study analyzing eight mental disorders (adding Tourette syndrome, anorexia nervosa, and obsessive–compulsive disorder) identified 11 pleiotropic loci. Among the most pleiotropic genes were RBFOX1, a splicing regulator involved in neuronal migration and synapse formation (33), and DCC, a protein critical for axonal guidance during neurodevelopment (34).

The latest update from the PGC cross-disorder working group extended this analysis to 11 psychiatric disorders, adding PTSD, anxiety, and problematic alcohol use to the previous eight (35). This large-scale study revealed substantial heterogeneity in genetic associations across psychiatric disorders, particularly between ADHD and ASD. While behavioral genetic research has often suggested strong overlap between the two, molecular analyses of common variants reveal a much weaker genetic correlation, challenging earlier assumptions. This discrepancy between behavioral and molecular findings underscores the limitations of cross-disorder generalizations and emphasizes the need for large-scale, integrated genomic studies to clarify distinct and shared pathways.

Additionally, a separate systematic review of ADHD-specific PRS revealed that these genetic variants are associated not only with ADHD diagnosis but also with a range of functionally relevant traits, including hyperactive/impulsive behaviors, impaired working memory, lower education attainment, reduced brain volume, higher body mass index, and decreased socioeconomic status (36). These findings highlight the dimensional nature of genetic risk and its broader impact on developmental, cognitive, and social outcomes.

#### 3.1.3 Mitochondrial DNA and X-linked inheritance

Emerging research on mitochondrial DNA (mtDNA) and X-linked inheritance has enriched the genetic discussions surrounding ADHD heritability, providing valuable maternal-specific insights. Specific mtDNA haplogroups, formed through sequential mutations that trace maternal ancestry and migration (37, 38), have demonstrated significant genetic correlations with ADHD (39, 40). For example, haplogroups B4, D4b, F, and HHV are positively associated with ADHD development, whereas haplogroups B5, K, and U are linked to protective effects (41).

These mtDNA transmission patterns reflect the mechanisms of X-linked inheritance, which include genes that escape X inactivation, random X inactivation in females, parental X imprinting, and the epigenetic regulation of X-linked genes (42). One proposed framework suggests that males with Klinefelter syndrome (47, XXY), who possess two X chromosomes, may exhibit phenotypic similarities to females with idiopathic ADHD, particularly with respect to inattentive symptoms (43). In support of this theory, studies indicate inhibitory control deficits in individuals with Turner syndrome (45, X) but not in those with Klinefelter syndrome, suggesting a potential neuroprotective effect conferred by the presence of two X chromosomes (43, 44).

While extensive research on maternal versus paternal genetic contributions specifically related to X inactivation remains limited, the differing prevalence rates of ADHD between males and females highlight the need for further investigation in this area. Despite the uncertainties surrounding the role of paternal genetics in this process, maternal genetics influences the presentation and severity of ADHD symptoms in offspring through both mitochondrial and X-linked mechanisms.

### 3.2 Epigenetic interactions

#### 3.2.1 Maternal gestational weight gain

Maternal gestational weight gain (GWG) has also been associated with an elevated risk of ADHD development in offspring through various hypothesized mechanisms during pregnancy (45). One key factor is the fat mass and obesity associated (FTO) gene, which has been shown to predispose mothers to obesity and excessive GWG (46). This genetic susceptibility, when passed to offspring, may contribute to ADHD risk (47).

Mechanistically, excessive GWG is linked to elevated levels of inflammatory and metabolic markers that cross into the fetal circulation, including monocyte chemoattractant protein-1, high-sensitivity C-reactive protein (CRP), and leptin (48). These factors are hypothesized to affect neuromodulatory processes in the developing brain.

Excessive GWG may also lead to increased leptin and insulin resistance, which may disrupt neuroendocrine signaling important for brain maturation (49). In addition, changes in the maternal and neonatal microbiome—driven by obesity or a high-fat diet—can influence fetal brain structure, cognitive function, mood, and behavior (50). Finally, GWG, either independently or in combination with a poor maternal diet, may lead to epigenetic changes in fetal gene expression, particularly in limbic reward circuits in the midbrain cortex (51). These epigenetic modifications could help explain the observed associations between maternal obesity and altered child behavior. Together, these findings suggest that excessive GWG is not only a marker of metabolic risk but also a potential epigenetic contributor to ADHD pathophysiology and may represent another opportunity for prenatal intervention and clinical guidance.

#### 3.2.2 Young maternal and paternal ages

Genetic predispositions to ADHD are influenced by various factors, including X chromosome number, mtDNA haplogroups, polygenic risk scores (PRS), and SNP-based heritability outlined previously. Recently, both young maternal and paternal ages have emerged with the promise to act on these genetic predispositions and significantly correlate to the intensification of ADHD genetic traits in children (52). While much of the existing research on young maternal age thus far has relied on primarily observational methods, a high-quality systematic review and meta-analysis from 2021 established a correlational link between lower parental age and an increased risk of ADHD in offspring (52). The study further indicated that parents under 20 years old face the highest risk, while age 31–35 years marks a point of diminishing risk, after which the likelihood of ADHD begins to rise again (52).

Children born to younger mothers specifically have been found to be nearly twice as likely to develop ADHD (53). However, this association may be confounded by factors such as higher ADHD prevalence among younger mothers, socioeconomic disadvantages, increased risk-taking behaviors, and greater rates of smoking, all of which are touched on in this review and described in more detail in the limitations section (53).

This increased ADHD risk is not exclusive to maternal age; this high-quality study showcased that children of either parent under 20 years old have higher chances of developing ADHD, with adjusted odds ratios (ORs) of 1.49 (95% CI: 1.19–1.87) for mothers and 1.75 (95% CI: 1.31–2.36) for fathers (52). Although these findings are statistically significant and come from a high-quality study, it is imperative to consider confounding factors related to paternal influences and genetics. Further investigation into variables like alcohol use, tobacco consumption, and poor nutrition is warranted (52, 53) to enhance understanding and inform systemic interventions targeting these issues.

#### 3.2.3 Maternal gene–psychopathology interactions

##### 3.2.3.1 Maternal ADHD and heightened harm avoidance

Research has shown that mothers of children with ADHD tend to exhibit greater levels of harm avoidance than parents of typically developing children do, particularly in lower socioeconomic status (SES) households (54). Harm avoidance includes traits such as anticipatory anxiety, fear of uncertainty, and behavioral inhibition, which are commonly associated with mood disorders and impaired emotional regulation (55, 56). Notably, mothers with significant ADHD traits often reported less improvement in their children’s ADHD symptoms following standard care interventions at the 12-month follow-up period. This has led to the development of a conceptual framework called the maternal ADHD resilience score (RS), which reflects mothers’ psychological adaptability under adversity. A lower RS may contribute to greater ADHD symptom severity in children, either directly or through diminished parenting effectiveness (57).

A nationwide study further supports this model, showing strong associations between maternal psychiatric diagnoses—including ADHD, depression, personality disorders, and substance use disorders—and ADHD in offspring, particularly among daughters (58). These associations are believed to stem from both genetic contributions, as outlined previously (e.g., mtDNA and X-linked transmission, alongside some paternal genetic impact), and environmental influences, such as maternal stress, perinatal hormonal changes, and parenting behaviors (58).

##### 3.2.3.2 Maternal and paternal depression

Among the maternal psychopathologies discussed, depression emerges as a significant predictor of long-term behavioral issues in children with ADHD. Longitudinal studies have demonstrated a strong association between maternal depression and conduct problems in children over an eight-year span, reinforcing earlier findings that link maternal depression to externalizing behaviors, particularly in offspring with ADHD (55, 56). The detrimental effects of maternal depression are thought to be partially mediated by disruptions in the quality of mother–child interactions. Depressed mothers tend to exhibit negative, disengaged, or unsupportive parenting behaviors, which can hinder a child’s emotional and cognitive development (55, 56). These disruptions can further exacerbate the expression of ADHD symptoms, especially in children who are already genetically predisposed to the disorder.

Crucially, the impact of maternal depression extends beyond the prenatal period, encompassing preconception, perinatal, and postnatal stages. This suggests that maternal mental health throughout the developmental timeline may have cumulative effects on the risk and severity of ADHD symptoms in offspring (59). Meta-analyses have specifically highlighted an association between maternal postpartum depression and an increased risk of developing ADHD later in life (60). Studies indicate that overall parental depression, including exposure during both the prenatal and postnatal periods, is linked to more than double the likelihood of ADHD in children (60).

While research is limited in scope regarding the full impact of paternal psychopathology, some inconsistencies aside from paternal depression—alongside paternal antisocial personality disorder—have been significantly correlated with offspring ADHD in a high-quality systematic review (60). This contributes to an overall elevated risk, compounded by maternal factors. Studies suggest that a key factor in this connection could be the coheritability of major depressive disorder and ADHD through a common genetic polymorphism, indicating a shared genetic risk that could be inherited from both parents. However, further research is needed to explore these complexities fully (32).

##### 3.2.3.3 Maternal temperament, pregnancy vs. postpartum depression, anxiety, and sleep disorders

In addition to maternal ADHD and depression, various other maternal psychopathologies significantly influence the risk of ADHD in offspring through both inherited genetic pathways and environmental factors. These psychopathologies include cyclothymic, anxious, irritable, and affective temperaments, alongside severe postpartum depression (59) and depression during pregnancy (59). All these maternal psychiatric conditions have been linked to an increased risk of ADHD across all subtypes in preschool-aged children. Furthermore, maternal conditions, including depression but also anxiety and sleep disorders, whether experienced before conception or during pregnancy, are associated with a heightened risk of ADHD in offspring (61, 62).

These psychopathologies are believed to exert their effects through shared genetic pathways, hormonal changes, and altered mother–infant interactions, thereby reinforcing the dual genetic and environmental impact of maternal mental health on child neurodevelopment. Exploring these additional dimensions of maternal mental health would provide valuable insights into their specific contributions to ADHD risk.

#### 3.2.4 Shared genetic loci associated with maternal education attainment, socioeconomic status and offspring ADHD

In alignment with the previously outlined shared genetic loci linked to comorbid conditions, a conjunctional analysis of ADHD and educational attainment (EA) examined how shared loci near KDM4A, PINK1, and MEF2C reveal a genetic connection between heightened ADHD phenotypes and maternal-specific decreased educational attainment (30, 63). A subsequent study utilizing significantly larger datasets from ADHD and EA genome-wide association studies (GWASs), along with additional intelligence data, expanded these findings by identifying 30 loci shared between ADHD, EA, and/or intelligence, including genes such as CALN1, FOXP1, FOXP2, PTPRF, and SORCS3 (30). Notably, while the general trend indicates negative genetic correlations between ADHD, EA, and intelligence, individual loci exhibited both concordant and discordant effects. These results suggest a complex interplay of gene–phenotype epigenetic interactions rather than simple inverse associations (64). This finding supports a model in which genetic variants have both disorder-specific and transdiagnostic effects and are influenced by functional pathways, developmental timing, and the environmental context. Moreover, polygenic risk scores (PRSs) for ADHD not only predict core symptoms but also extend to traits such as impaired working memory, lower educational attainment, and reduced socioeconomic status, further underscoring the multifaceted phenotypic impact of these variants across various domains.

Research examining the intergenerational transmission of educational attainment (EA) has revealed compelling associations between maternal EA and offspring ADHD outcomes, extending beyond mere genetic and epigenetic interactions. Children of parents with lower EA are statistically more likely to have lower socioeconomic status (SES) (65). A systematic review evaluated the correlation between low SES and ADHD in children and revealed that children from low-SES families are, on average, 1.85 to 2.21 times more likely to have ADHD than their peers are (66). These children often face increased ADHD symptomatology and academic challenges, with diminished cognitive stimulation, support, and resources at home contributing to these adverse outcomes (65).

One plausible explanation for this correlation is that children of mothers with higher EA, who frequently also have increased SES, are better positioned to seek and adhere to ADHD treatments (66). Mothers with higher EA and SES are generally more adept at recognizing symptoms, pursuing diagnostic evaluations, and accessing interventions for their children. This advantage stems partly from their greater confidence in advocating for their children’s needs, along with higher health literacy and financial access to treatment options (65, 67, 68). Conversely, the heightened incidence of ADHD diagnoses among children from low SES households may be confounded by factors such as parental mental health and maternal smoking during pregnancy (66).

Research indicates that while genetic and environmental factors contribute to ADHD risk, higher maternal EA and income levels are consistently linked to better developmental outcomes (69), including a reduced likelihood of ADHD diagnosis between ages 9 and 11 (70). The landmark 1999 Multimodal Treatment of ADHD (MTA) study reinforces this finding by demonstrating that children with the combined subtype of ADHD from socioeconomically disadvantaged backgrounds experienced poorer long-term developmental outcomes (14, 15). However, the study was limited by insufficient control over confounding variables, and follow-up results varied, highlighting the complexity of SES influences. Nevertheless, these findings emphasize the importance of considering socioeconomic factors, such as maternal education and income, as both independent moderators of ADHD and indirect indicators of genetic predisposition. Addressing these social determinants—through enhanced education, access to resources, or support for maternal mental health—may offer promising pathways to improving outcomes for at-risk children.

#### 3.2.5 Postnatal household chaos, maternal hostility and maltreatment

Environmental risk factors emerging during the postnatal child-rearing period also play a critical role in shaping ADHD outcomes. Children raised in chaotic household environments—characterized by high levels of disorganization, unpredictability, and noise—are significantly more likely to display ADHD symptoms (71). This association is especially pronounced when both the child and the mother have elevated ADHD PRSs, highlighting the interplay between genetic vulnerability and environmental stressors (72). Importantly, this relationship persists regardless of whether chaos is consciously perceived, supporting the concept of gene–environment correlation (rGE). In this context, rGE helps explain how genetically influenced traits may interact with or evoke certain environmental conditions. Two key forms are particularly relevant here: evocative rGE, in which a child’s genetically driven behaviors (e.g., impulsivity) may elicit negative or hostile parenting responses. In passive rGE, shared genetic factors may influence both the parent’s behavior (e.g., hostility) and the child’s ADHD symptoms, leading to apparent environmental effects that are partially genetically mediated (73).

These rGE dynamics have been observed not only in biological families but also in adoptive mother–child relationships, illustrating that both genetic and environmental factors can heighten hostility and negativity from mothers, regardless of biological ties. In this context, household chaos and maternal hostility may not merely coexist with ADHD; they can interact epigenetically with a child’s genetic predispositions, exacerbating symptom expression. This notion is further supported by a robust meta-analysis focusing on parental maltreatment, particularly emphasizing maternal influence. The findings indicate that children who experience maltreatment and abuse—often stemming from maternal hostility—are seven times more likely to exhibit ADHD symptoms than control groups are (74). This perspective reinforces the understanding that maternal genetic predispositions and environmental behaviors are not isolated factors; rather, they are interconnected forces that significantly shape neurodevelopmental outcomes in children.

#### 3.2.6 Prenatal maternal stress

Another significant modifiable factor is prenatal maternal stress (PNMS), which includes experiences such as relationship instability, residential moves, and divorce. These stressors negatively affect maternal functioning during pregnancy and, in turn, influence neurodevelopmental outcomes in offspring, including ADHD risk (75).

While some inconsistencies exist across studies, the fetal reprogramming hypothesis remains a leading explanatory model. It has been proposed that excess maternal cortisol during pregnancy alters the intrauterine environment leading to changes in the fetal brain that impact emotional regulation and behavior postnatally (76). Recent expanded epigenetic research on this model, suggesting that DNA methylation patterns may serve as a molecular mechanism linking prenatal stress to ADHD expression.

A 2020 meta-analysis examined DNA methylation at birth by analyzing cord blood, correlating it with recurrent ADHD symptoms in school-aged children (77). The study not only established that DNA methylation at birth is associated with ADHD, identifying nine CpGs, including ERC2 and CREB5, that predict later ADHD symptoms (p < 0.0000001) but also revealed that greater maternal prenatal stress was linked to increased DNA methylation in cord blood. Specifically, each standard deviation (SD) increase in prenatal stress corresponded to a 0.2 SD increase in childhood ADHD symptoms (SE = 0.03, p = 0.00000000002, n = 1121), providing strong statistical evidence for the relationship between prenatal maternal stress (PNMS) and ADHD-related outcomes (77).

These findings reinforce the notion that prenatal stress can induce enduring changes in gene expression, particularly in neurodevelopmental pathways associated with attention and behavior. This hypothesis is further supported by additional research connecting PNMS to a wider array of childhood developmental issues, including difficulties in emotional and behavioral regulation, delayed language development, cognitive challenges, mixed handedness, and an increased risk for neurodevelopmental disorders such as schizophrenia (76, 78–80).

Collectively, these findings underscore the significant impact of both risk and protective factors in maternal stress on developmental outcomes and treatment responses in children with ADHD, highlighting opportunities for targeted psychosocial interventions. While further studies are needed to validate the utility of methylation variation as a biomarker for prenatal maternal stress and clarify its role in causal pathways, doing so could enable a shift from primarily correlational findings to more definitive causal evidence.

#### 3.2.7 Maternal parenting and attachment style

As awareness of the importance of psychosocial interventions grows, the targeting of maternal parenting strategies is increasingly recognized as a vital factor in mitigating symptom expression in children with ADHD. Strengthening the quality of mother–child interactions has been shown to reduce symptom severity and enhance behavioral outcomes. Research indicates that positive parenting and secure parent–child attachments can act as buffers against the development and progression of maladaptive behaviors associated with ADHD (81). For example, mothers who consistently offer praise and maintain a positive demeanor during structured interactions tend to have children who exhibit fewer conduct problems and lower overall deviance (82). These protective effects are particularly pronounced in high-demand situations, where children face challenges related to inattention, hyperactivity, or impulsivity (83–85). This illustrates that early positive parenting serves as a crucial factor in reducing the likelihood of escalating conduct issues in children diagnosed with ADHD.

Significantly, a recent 2022 meta–analysis reinforced these findings, revealing statistically significant associations between parenting practices and subsequent ADHD symptoms and diagnoses (74). While the number of high–quality studies on this topic remains limited, poorer interaction quality, maltreatment or abuse, single parenting, and excessive media exposure have been linked to an increased likelihood of ADHD symptoms and diagnoses (74). These insights suggest opportunities for prevention through public health initiatives that address maltreatment and promote positive parenting strategies. However, further high–quality research is needed to explore these associations in detail, particularly with respect to specific positive parenting styles and attachment dynamics.

The protective role of parenting is further emphasized in Gerald Patterson’s coercive family process theory, which posits that ineffective early interactions with challenging children may lead to either maternal withdrawal or hostility, ultimately exacerbating maladaptive behaviors (86). This bidirectional relationship between hostile parenting and child ADHD symptoms highlights how parental behaviors can both respond to and reinforce symptom expression. Notably, maternal hostility has been linked to poorer academic outcomes, particularly in mathematics (87), among children with ADHD, likely reflecting the impact of ADHD symptoms on task persistence and cognitive flexibility—skills that are especially critical in math. Interestingly, this association appears stronger than the previously reported connection between ADHD and reading difficulties (87), potentially due to the protective influence of higher socioeconomic status (SES) households, which often provide greater literacy support and stimulation. Together, these findings underscore the essential role of parenting–focused interventions as a complement to medical treatment, particularly in shaping early behavioral patterns and mitigating the long–term academic and emotional impacts of ADHD.

### 3.3 Maternal environmental exposures

#### 3.3.1 Acetaminophen exposure *in utero*

While gene–epigenetic mechanisms have been previously discussed and are important, emerging environmental pathophysiological processes provide new insights into the relationship between maternal exposure and ADHD in children. These insights may help explain not only the prevalence of ADHD but also the severity of symptoms, offering a broader understanding that goes beyond genetic–epigenetic studies.

During the pregnancy period, acetaminophen use has recently emerged as a significant concern. Although the literature varies in terms of exposure duration and outcome assessment, several high–quality studies have linked acetaminophen use during pregnancy with an increased risk of ADHD in offspring (81, 88). This association is particularly pronounced when acetaminophen is taken during the third trimester or for more than 28 days (89).

While the exact biological mechanisms are not fully understood, one prevailing hypothesis is that prenatal acetaminophen exposure may disrupt neurodevelopment via several potential pathways, including the formation of the toxic metabolite N–acetyl–p–benzoquinone imine (NAPQI), oxidative stress from inflammation–induced immune activation, altered expression in brain–derived neurotrophic factor (BDNF), endocannabinoid dysfunction, cyclooxygenase–2 (COX–2) inhibition, and endocrine disruption (90). This theory is supported by evidence that acetaminophen readily crosses the placenta and the blood–brain barrier (91–93), raising concerns about its neurodevelopmental safety during critical windows of fetal brain maturation.

#### 3.3.2 Maternal diet and vitamin D deficiency

Within the pre– and perinatal periods, low vitamin D levels have emerged as a significant biomarker for ADHD risk (90). While a high–quality umbrella review from 2020 suggested that low vitamin D levels before and during pregnancy contributed to ADHD in offspring, the evidence at the time was limited in both strength and mechanistic explanation (90). A meta–analysis from 2024 (94) revealed emerging evidence of a contribution of both vitamin D deficiency and low intake of omega–3 fatty acids, particularly eicosapentaenoic acid (EPA), to the development of ADHD in offspring. Improved maternal DASH scores (dietary approaches to stopping hypertension), reflecting a higher–quality diet, were found to be correlated with a reduced risk of borderline to clinical–range symptoms related to ADHD. Specifically, these scores were associated with a decrease in depressive and anxiety symptoms (OR 0.97, 95% CI: 0.95–0.99), aggressive behavior symptoms (OR 0.97, 95% CI: 0.94–0.99), and attention–deficit hyperactivity disorder symptoms (OR 0.97, 95% CI: 0.95–0.98) (94).

Improved maternal DASH scores (dietary approaches to stopping hypertension), reflecting a higher–quality diet, were found to be correlated with a reduced risk of borderline to clinical–range symptoms related to ADHD. Specifically, these scores were associated with a decrease in depressive and anxiety symptoms (OR 0.97, 95% CI: 0.95–0.99), aggressive behavior symptoms (OR 0.97, 95% CI: 0.94–0.99), and attention–deficit hyperactivity disorder symptoms (OR 0.97, 95% CI: 0.95–0.98). Conversely, a one–unit increase in energy–adjusted dietary inflammatory index (E–DII) scores, which indicates a more proinflammatory diet, was linked to a 7% increased risk of all three areas of concern: anxiety and depression (OR 1.07, 95% CI: 1.03–1.11), attention–deficit hyperactivity disorder (OR 1.07, 95% CI: 1.01–1.13), and aggressive behavior (OR 1.07, 95% CI: 1.02–1.13).

While the study investigated a wider range of outcomes, including ASD, anxiety, and depression, the findings specifically related to ADHD were statistically significant, thus, these data and studies were included in this review. A negative correlation was observed between adherence to a DASH diet, which indicates better dietary quality, and reduced ADHD symptoms in offspring. In contrast, there was a positive correlation between a higher E–DII score, which signifies a more proinflammatory diet, and increased ADHD symptoms. These results bolster the argument for exploring dietary interventions during pregnancy, such as vitamin D and omega–3 supplements, as low–cost and accessible adjuncts to traditional ADHD pharmacotherapies.

#### 3.3.3 Perinatal toxic metal exposure

Within the perinatal period, previous lower–quality observational studies suggested that elevated lead levels are associated with ADHD–related symptoms, potentially by disrupting fetal brain development (95), since lead can readily cross the placenta and has been linked to neurodevelopmental deficits (95–98). These findings are strongly supported by a comprehensive umbrella review of 63 meta–analyses, which revealed a statistically significant association between higher blood lead levels and ADHD, with an odds ratio (OR) of 1.74 (95% CI: 1.39–2.18) (90, 99). This relationship was graded as highly suggestive (Class II), with no evidence of small–study effects or excess significance bias. The association also held under a 10% credibility ceiling and was based on data from large, well–conducted studies. This robust association confirms that elevated lead exposure is not only linked to general neurodevelopmental deficits but also may contribute specifically to at least one subtype of ADHD. Owing to this significance, it becomes imperative for pregnant mothers to be aware of the possible ways to be exposed to lead, including contaminated food or water, lead–based pain, secondhand smoke, and air pollutants (100).

Another metal of particular concern is cadmium, predominantly in the context of maternal tobacco use (90, 101, 102). This connection is especially important considering polygenic risk scores (PRSs). As discussed previously, elevated PRSs for ADHD are associated not only with increased risk of the disorder itself but also with traits commonly linked to comorbid psychiatric conditions, including heightened risk–taking behavior (103). These behavioral tendencies can influence maternal lifestyle choices—such as smoking during pregnancy—which increases fetal exposure to cadmium (103). Furthermore, young maternal age is linked to higher rates of smoking and other risk–taking behaviors (52, 53), as previously discussed in this review, serving as confounding factors in the correlation between young parental age (less than 20 years) and offspring ADHD. Collectively, these findings highlight the complex interplay between maternal genetic predisposition and modifiable exposures, illustrating how inherited risk can shape behavioral pathways that heighten developmental vulnerability in children. Additionally, the distinct roles of young fathers versus mothers, smoking, and elevated risk–taking behaviors—along with their association with lower socioeconomic status—present potential confounding effects. It is essential to better study these factors in isolation where possible to clarify their individual contributions.

Furthermore, a 2024 study employing a two–sample Mendelian randomization approach provided stronger evidence for causality concerning maternal smoking and fetal cadmium exposure (104). This inverse variance weighted (IVW) analysis revealed that maternal smoking around birth significantly increased the risk of ADHD in children, with notable statistical correlations: childhood ADHD was associated with an odds ratio (OR) of 3.63 (95% CI: 2.25–5.87, p = 0.000000131), late–diagnosed ADHD had an OR of 2.99 (95% CI: 1.74–5.14, p = 0.0000733), and persistent ADHD was linked to an OR of 4.77 (95% CI: 1.88–12.14, p = 0.00103).

This study revealed a causal, not merely correlative, relationship between increased prenatal cadmium exposure (from smoking) and an increased prevalence of ADHD in offspring. In contrast, subnormal magnesium levels have been associated with increased ADHD incidence (105), possibly due to magnesium’s role in fetal synaptic plasticity, as well as dopaminergic and serotonergic signaling (105, 106).

While cadmium has yet to be definitively classified as a developmental neurotoxicant (107), these findings underscore the need to raise awareness around modifiable prenatal exposures—including smoking–related cadmium risk and nutritional deficiencies—and their cumulative impact on long–term neurodevelopment. With growing evidence supporting not only correlation but also causality, these insights provide a critical foundation for developing targeted prenatal interventions aimed at reducing ADHD risk in genetically susceptible offspring.

### 3.4 Clinical manifestations

#### 3.4.1 Clinical heterogeneity of theta and beta waves in ADHD

ADHD is a clinically heterogeneous disorder lacking a singular pathophysiological etiology and involving multiple neurodevelopmental pathways (20). This complexity likely contributes to the estimated 74% heritability of the condition (3). Although a definitive pathophysiological mechanism for ADHD has not been established, various testing modalities over the past two decades have shown promise in elucidating its neural underpinnings. Electrophysiology studies have shown increased theta and beta wave activity in frontal brain regions in children and adolescents with ADHD. Notably, beta wave activity appears to be most elevated in the combined ADHD subtype (20, 108–110). These abnormal oscillatory patterns may serve as potential biomarkers of ADHD subtype differentiation and offer insight into the neurocognitive variability observed across affected individuals.

#### 3.4.2 Blood flow variations in the dorsolateral prefrontal cortex

Brain imaging techniques, specifically single–photon emission computed tomography (SPECT) (111, 112), have provided continued insights into variations in brain activity profiles in adults with ADHD, as originally shown in functional magnetic resonance imaging (fMRI) studies. SPECT imaging has shown increased blood flow in the medial anterior prefrontal cortex, the left anterior temporal lobe, and the right insular cortex in adults with ADHD than in neurotypical controls (113). In contrast, studies of children with ADHD have revealed decreased perfusion in the frontal (113) and orbitofrontal cortices (28, 114) and, specifically, a pattern of reduced cerebral blood flow in the left dorsolateral prefrontal cortex (DLPFC) alongside elevated flow in the right DLPFC. This hemispheric imbalance has been associated with increased symptom severity, particularly in domains related to impulsivity, attention, and decision–making (115). Additionally, studies examining ADHD and learning disability comorbidity have highlighted reduced temporal lobe perfusion (3, 116).

The DLPFC is crucial for executive control functions, including task switching, task–set reconfiguration, inhibition and interference control, planning, and working memory (117–120). More specifically, the left DLPFC supports target–directed perception, attention allocation, memory management, and decision–making, whereas the right DLPFC is associated with slower, more deliberate processing and temporal regulation of behavior (32). Thus, the observed hypoactivity in the left DLPFC and hyperactivity in the right DLPFC may explain the core features of ADHD—impaired memory management, attention, decision–making, and impulse control—and serve as a predictor of clinical symptom severity.

#### 3.4.3 Neuroanatomy of the ADHD–ASD functioning brain

While advancements in ADHD imaging are substantial, it is equally important to consider findings that explore neuroanatomical overlap with autism spectrum disorder owing to the high comorbidity rate with ADHD. A recent 2023 study utilizing data provided by the EU–AIMS LEAP project, which is a European multicenter study on stratification biomarkers for autism (121, 122), demonstrated that the neuroanatomy of ASD is significantly modulated by the presence of cooccurring ADHD (122). This interaction may be driven by atypical gene expression, which contributes to distinct cortical structure differences in individuals with both diagnoses (122).

Neuroimaging revealed changes in cortical thickness (CT) and surface area (SA) across several cortical regions, depending on the presence of comorbid ADHD or ASD. For example, PRSs for ADHD have been associated with a smaller caudate volume in children (N = 1139) and reduced total brain volume in children and adolescents with and without ADHD (N = 511) (77). Additionally, specific cortical regions where ADHD–ASD interactions were observed include the right temporal gyrus and right cingulate gyrus—both commonly altered in individuals with isolated ADHD—as well as the left precentral gyrus (for CT) and the right dorsolateral prefrontal gyrus (for SA) (122). These findings reinforce the idea that ADHD and ASD are not fully distinct neurodevelopmental conditions. Instead, when cooccurring, they may produce unique neuroanatomical signatures that differ from either condition alone. Given these interactions, future neuroimaging research on ADHD should continue to account for ASD comorbidity, particularly considering its influence on brain structure, function, and symptom expression.

Taken together, these imaging–based observations—ranging from heterogeneity in frontal theta and beta wave activity to asymmetrical perfusion patterns in the dorsolateral prefrontal cortex (DLPFC) to cortical structural differences in comorbid ADHD–ASD presentations—offer promising avenues for improving diagnostic precision and clinical stratification. With further validation, these neurobiological markers could support more structured, objective diagnostic tools, enable earlier identification of high–risk children, and inform individualized treatment planning. This neuroanatomical insight further emphasizes the extensive impact of ADHD on cognitive function, behavioral regulation, and overall quality of life. This underscores the necessity of recognizing comorbid conditions—such as ASD—alongside the maternal and certain paternal factors discussed in this review when developing effective interventions.

## 4 Limitations

The primary challenge facing contemporary maternal genome–wide association studies (GWASs) is their limited sample sizes, especially concerning comorbid conditions associated with maternal ADHD, the variability of risk alleles, mitochondrial DNA (mtDNA) variations, and X–linked inactivation. To uncover additional genome–wide significant variants, significantly larger sample sizes are imperative, this would increase generalizability, improve clinical applicability, and increase statistical power. Such advancements would not only strengthen emerging mtDNA research but also enable a clearer understanding of the distinct genetic contributions from mothers versus fathers concerning X inactivation, given the differing prevalence of ADHD across genders.

Moreover, prevalent comorbid maternal and offspring mental health issues, particularly depression and autism spectrum disorder (ASD) (122), exhibit shared single–nucleotide polymorphisms (SNPs) and similar brain region interactions, as evidenced by a limited number of studies (122). This intersection underscores the urgent need for further inquiry to precisely delineate the diagnoses of these intertwined conditions. Unfortunately, prior research has inadequately separated the specific risk alleles associated with ADHD from those relevant to common comorbidities. Closing this gap is vital for exploring potential causal relationships between these genetic factors, ADHD, and the ensuing development of psychopathologies in both mothers and their children.

In exploring epigenetic factors and confounding variables, complex interactions exist among socioeconomic status, less recognized maternal psychopathologies, rare genetic variants, and paternal epigenetic influences (30). Future research should focus on these rare genetic variants linked to ADHD while controlling for factors such as socioeconomic status and variables associated with young maternal age (51).

Notably, there is a scarcity of recent, high–quality studies investigating the confounding effects on young parental age and child ADHD – notably of stressors, poor nutrition, and smoking from both maternal and paternal perspectives (52) – particularly regarding the increased offspring ADHD risk for mothers under 20 years old and the negative correlation for mothers aged 31–35. While these confounders could impact internal validity, we chose to include this discussion to highlight the novelty of the association whether it is truly due to parental age, from these intermediate modifiers, or all the above. We encourage future research to build upon this foundation, enhancing our understanding to better inform systemic interventions, particularly in areas like diet and smoking cessation.

This review also recognizes the multitude of maternal environmental factors that could influence ADHD risk, however, not all these factors were examined owing to the emphasis on critically evaluating the available evidence and concentrating on the most significant correlations. For example, existing observational studies have indicated a potential link between maternal exposure to escitalopram and ADHD, however, a higher–quality systematic review refuted this association, leading to its exclusion from our analysis (123). Notably, findings from a recent umbrella review (90) highlighted several factors with robust evidence of associations (class I), such as maternal pre–pregnancy obesity (OR 1.63, 95% CI 1.49–1.77), childhood eczema (OR 1.31, CI 1.20–1.44), hypertensive disorders during pregnancy (OR 1.29, 1.22–1.36), preeclampsia (OR 1.28, CI 1.21–1.35), and acetaminophen exposure during pregnancy (RR 1.25, 95% CI 1.17–1.34). Additionally, factors with suggestive evidence (class II) included maternal smoking during pregnancy (OR 1.60, 95% CI 1.45–1.76), childhood asthma (OR 1.51, CI 1.4–1.63), maternal pre–pregnancy overweight (OR 1.28, CI 1.21–1.35), and diminished serum vitamin D levels (WMD −6·93, 95% CI −9·34–−4·51) (90).

Upon thorough investigation of updated meta–analyses and systematic reviews, the association between maternal pre–pregnancy overweight/obesity and offspring ADHD was largely attributed to familial confounding rather than a direct impact and, thus, was not extensively emphasized in this review (124). On the other hand, the focus was on weight gain, particularly during pregnancy. A recent meta–analysis highlighted that although observational studies have linked maternal obesity to negative outcomes for offspring, there is still a lack of strong evidence from Mendelian randomization studies (125).

Observational studies of preeclampsia and hypertensive disorders have suggested strong associations with ADHD. Recent studies revealed no significant correlation between intrauterine exposure to preeclampsia and ADHD risk, despite noted associations with ASD and epilepsy (90). These findings complicate the relationship between preeclampsia and ADHD because of the inability to disentangle effects stemming from preterm birth, a confounding variable (90, 125–128).

Although the childhood condition of eczema patients is significantly correlated with ADHD, this review did not address it, as studies frequently conflated maternal influence with the comorbidities observed in children (129, 130). Furthermore, additional factors, such as perinatal hypoxic conditions, yielded low–level associations (class IV evidence) and were not explored further. Despite the exploration of fetal alcohol exposure, findings have been deemed insignificant enough to warrant exclusion from this review (131).

This review has focused predominantly on maternal genetic, epigenetic, and environmental factors but has not thoroughly addressed paternal contributions. Importantly, most existing shared gene studies do not definitively isolate whether the evaluated genes are solely inherited from the mother, indicating a potential role for paternal factors as well. Notably, significant correlations have emerged linking paternal smoking, paternal depression, and young paternal age to the onset and severity of ADHD in children (60). These findings highlight the necessity for more in–depth studies on paternal influences.

However, existing high–quality research predominantly underscores maternal influences—whether gene–environment or gene–epigenetic influences—showing a more consistent role in ADHD development than paternal factors do. Inconsistent findings concerning paternal influence include a study revealing that, among children diagnosed with ADHD, paternal antisocial personality disorder, rather than paternal ADHD, was correlated with conduct problems through negative parenting behaviors (60). Addressing confounding variables in paternal influences is challenging, as they are often less controllable than maternal–fetal interactions are, complicating our understanding of ADHD etiology (57, 58). Nonetheless, it is essential to acknowledge that paternal contributions should not be overlooked, as highlighted by insightful reviewer feedback during the peer–review process. Future research should systematically investigate paternal factors in conjunction with maternal influences to yield a more comprehensive understanding of ADHD risk.

Moreover, this review faced challenges in locating studies specifically examining paternal genetic inheritance related to ADHD, indicating a significant gap in the literature. Recent high–quality systematic studies have explored the effects of paternal environmental and epigenetic impacts, such as alcohol, tobacco, caffeine use, and physical activity, on the mental health of offspring, including ADHD (132). Research indicates that paternal smoking during pregnancy may significantly increase the likelihood of ADHD in offspring (OR 1.42), and other studies suggest that paternal factors may be more closely linked to externalizing behaviors in children (133). However, many of these findings stem from older, predominantly observational studies that raise concerns about research consistency and the reliability of the outcomes. This underscores the urgent need for further exploration of paternal environmental effects during the pre–pregnancy, perinatal, prenatal, and post–pregnancy periods to establish their causal implications.

Another limitation lies in the population–specific focus of the selected studies, such as those involving Norwegian or Korean mothers, which may affect the generalizability of the results. Although most reviewed studies included participants of European ancestry and English–speaking backgrounds within the U.S. context, this diversity highlights the multifaceted nature of the subject under examination. While potential generalizability limitations must be acknowledged, it is also crucial to recognize that foundational knowledge in this emerging field often necessitates observational studies.

Interestingly, the theme of positive maternal parenting was the only area in this review lacking robust support from high–quality studies. Notably, while a meta–analysis indicated that both maternal and paternal ADHD symptoms accounted for a small percentage of variance in parenting styles, it failed to comprehensively examine the direct relationship between these parenting styles and ADHD in offspring (134). This omission highlights a significant gap, suggesting the need for more focused research on how parenting styles may influence the propensity for ADHD in children. The limited number of studies in this domain emphasizes the need for further investigation into the connections between parenting styles and ADHD outcomes. In summary, while maternal factors dominate this review, future studies must incorporate a more balanced examination of both maternal and paternal contributions to ADHD, aiming for a well–rounded understanding of the complex interplay of genetic, epigenetic, and environmental influences that contribute to this condition.

Examples of maternal interventions that effectively reduce the prevalence or severity of ADHD symptoms in offspring remain limited. In our review, we highlight a systematic review and meta–analysis by Li et al. (2019), which investigated the impact of preconception and prenatal nutrition on autism spectrum disorder (ASD) and ADHD. While a clear inverse association with ASD has been established, the evidence regarding ADHD has been inconclusive. Despite the genetic overlap between these two conditions, the scarcity of targeted research on maternal interventions for ADHD underscores the urgent need for focused studies in this area (135).

To address potential biases, we also examined paternal interventions aimed at mitigating ADHD in children and adolescents. A comprehensive meta–analysis and umbrella review by Turk et al. (2023) revealed a significant lack of robust evidence supporting effective combination treatments for ADHD. However, the study did demonstrate strong correlations between parental interventions—both maternal and paternal—and improved ADHD ratings in offspring, showing significant effects from pharmacological treatments (SMD = 0.67, 95% CI 0.60 to 0.74) and teacher ratings (SMD = 0.68, 95% CI 0.54 to 0.82) as well as psychological interventions (SMD = 0.42, 95% CI 0.33 to 0.52) for parents and (SMD = 0.25, 95% CI 0.12 to 0.38) for teacher–reported symptoms. Nonetheless, specific effect size data for combination therapies were absent from this study (136).

Additionally, a systematic review examining parenting interventions designed to enhance father engagement reported disappointing results. Research has revealed that efforts to promote father involvement in treatment initiation resulted in low engagement rates, with 58% of studies either not reporting father engagement or showing engagement levels below 50% (137). This highlights the need for future research to rigorously evaluate the effectiveness of interventions across the preconception, prenatal, and postnatal periods, clarifying their potential impact on ADHD risk. Moreover, emphasizing the maternal–specific factors discussed in this review could unveil promising pathways for developing more effective pharmacological and psychological combination treatments for children with ADHD, helping to elucidate causal mechanisms.

Although definitive causality between maternal factors and ADHD in offspring remains underexplored—maternal cadmium exposure through smoking being the only clearly defined causal factor in this review—other significant associations identified, such as maternal genetics, environmental influences, mental health diagnoses, and protective factors, present meaningful opportunities for targeted preemptive interventions in clinical settings. This study highlights the intricate interplay of maternal influences on ADHD while acknowledging several areas warranting further research. Key gaps include the need for larger, more diverse population samples, balanced paternal influence studies, and a deeper understanding of atypical ADHD risk variants, maternal gene–psychopathology interactions, young maternal age, antidepressant use, harmful parenting behaviors, and socioeconomic confounding. By comprehensively understanding these interconnections, we can enhance our ability to proactively mitigate ADHD manifestations at both the individual and societal levels, particularly by effectively addressing maladaptive behaviors associated with comorbid conditions.

## 5 Conclusion

Numerous maternal–specific factors contribute to both the exacerbation and reduction in the prevalence and severity of ADHD in offspring. These factors primarily encompass maternal genetics with some paternal genetic influence, maternal psychopathologies, epigenetic effects, the maternal environment, and clinical manifestations. The interplay among these factors emphasizes the intricate nature of ADHD and underscores the crucial role that mothers play—not only in the emergence of the condition in their children but also in the potential to moderate the extent of its detrimental effects.

Given that parents have the most consistent influence on child development, screening for maternal risk factors—even before ADHD symptoms emerge—becomes critical. By implementing supportive programs for at–risk mothers, which include not only pharmacological interventions for maternal mental health diagnoses but also providing resources and parenting interventions for psychological interventions, there is a potential to protect against the progression of ADHD symptoms in children. This review highlights the urgent need for more research into these interventions, especially considering the increasing incidence of ADHD among children, advocating for increased funding and support to better address these issues from the perspective of social determinants of health.
